# Evaluation of survival of patients with hepatocellular carcinoma: A comparative analysis of prognostic systems

**DOI:** 10.1371/journal.pone.0194922

**Published:** 2018-04-04

**Authors:** R. K. Tannus, S. R. Almeida-Carvalho, C. A. Loureiro-Matos, A. Miziara-Gonzalez, A. A. Salzedas-Netto, D. Szejnfeld, G. D'Ippolito, V. Pereira-Lanzoni, I. Souza-Silva

**Affiliations:** 1 Department of Gastroenterology, Hepatology Unit, Federal University of Sao Paulo (Unifesp), Sao Paulo, SP, Brazil; 2 Department of Surgery, Liver Transplant Unit, Federal University of Sao Paulo (Unifesp), Sao Paulo, SP, Brazil; 3 Department of Pediatric Surgery, Federal University of Sao Paulo (Unifesp), Sao Paulo, SP, Brazil; 4 Department of Diagnostic Radiology, Federal University of Sao Paulo (Unifesp), Sao Paulo, SP, Brazil; 5 Department of Diagnostic Pathology, Federal University of Sao Paulo (Unifesp), Sao Paulo, SP, Brazil; Universita degli Studi di Pisa, ITALY

## Abstract

**Background and aim:**

There are several prognostic systems that address different aspects of the patient and the tumour and can guide the management of patients with hepatocellular carcinoma (HCC). This study aimed to evaluate and compare the eight staging systems for a group of patients in a public service in Brazil.

**Methods:**

Patients with HCC were retrospectively analysed between 2000 and 2012. The prognostic systems Okuda, The Cancer of the Liver Italian Program (CLIP), the Chinese University Prognostic Index (CUPI), Groupe d'Etude et de Traitément du Carcinome Hepatocellulaire (GRETCH), the modified TNM-based Japan Integrated Score (JIS) combined with alpha-fetoprotein and Child-Turcotte-Pugh (CTP), the TNM system, and the Barcelona Clinic Liver Cancer Classification (BCLC) were applied to these patients and compared through model fit measurements, likelihood scores, and the Akaike Information Criterion (AIC).

**Results:**

A total of 247 patients were studied. The average survival time was 60 months. The TNM, Okuda, CLIP, GRETCH, modified JIS, and BCLC systems were well correlated with one another and individually important to the prediction of survival among the patients studied. However, in the statistical analysis, the CUPI delivered the best predictive performance (AIC = 566; log-likelihood = -281,240).

**Conclusion:**

Although the CUPI system was demonstrated to be the most appropriate HCC staging system for the studied population, the choice of an ideal system is a controversial subject, and future studies with larger numbers of patients are necessary for the validation of the CUPI system as the method of choice for other populations.

## Introduction

Hepatocellular carcinoma (HCC) is responsible for approximately 250,000 to 1 million deaths per year worldwide [1–3]. The high mortality of HCC is the result of multiple factors, including inefficient screening, a lack of specialized treatment (such as liver transplantation or ablative therapies) in some regions, and late diagnosis in patients with advanced tumours and often low hepatic functional reserves [4–8]. To better evaluate the chances of survival of these patients and to standardize therapeutic indications, several prognostic evaluation systems have been developed by various groups worldwide.

The Okuda system [9] accounts for aspects of both the tumour (size ≤ or > 50% of the entire liver) and clinical aspects (presence or absence of ascites, serum albumin and serum bilirubin levels). The Cancer of the Liver Italian Program (CLIP) [10] includes the number of nodes, alpha-fetoprotein (AFP) level (>400ng/dL), portal vein thrombosis and Child-Turcotte-Pugh (CTP), classification. The Chinese University Prognostic Index (CUPI) [11] analyses tumour-node-metastasis (TNM) stage and clinical and laboratory data including bilirubin, alkaline phosphatase and AFP level (>500ng/mL). The *Groupe d'Etude et de Traitément du Carcinome Hepatocellulaire* (GRETCH) [12] also addresses laboratory data such as bilirubin, alkaline phosphatase and AFP levels (>35ng/mL) and includes radiological data (portal vein obstruction). The modified TNM-based Japan Integrated Score (JIS) [13] combined the AFP >400ng/dL and the CTP [14]. The TNM scale [15] accounts for aspects of the tumour (size and spread to lymph nodes/distant sites). The Barcelona Clinic Liver Cancer Classification (BCLC) system [16] addresses the aspects of a tumour (size, extension and presence of portal vein thrombosis/extrahepatic metastasis), CTP, performance status, cancer symptoms and treatments options (Table 1). Among these systems, the CUPI was validated in a cohort consisting mostly of hepatitis virus B-infected patients, and CLIP and JIS scores are primarily designed for hepatitis C patients.

**Table 1:** Description of HCC staging systems.

System	Okuda	CLIP	GRETCH	CUPI	TNM	JISTNMAFP	BCLC
**Tumour Size**	x	x		x	x	x	x
**Nodes**				x	x	x	
**Metastases**				x	x	x	x
**Portal vein thrombosis**		x	x				x
**Alpha-fetoprotein**		x	x	x		x	
**Child-Turcotte-Pugh**		x				x	x
**Albumin**	x						
**Total bilirubin**	x		x	x			x
**Alkaline phosphatase**			x	x			
**Ascites**	x			x			
**Performance status**			x				x

It is notable that these systems are able to address various aspects of the patient and the tumour in different populations that may differ in the aetiology of liver disease, which could hinder the homogeneous application of these systems across different geographical regions. Thus, this study aimed to evaluate and compare the efficacy of each staging system in a group of patients diagnosed with HCC in a specialized public service in Brazil.

## Materials and methods

Patients with HCC treated at the liver transplantation section of the surgical gastroenterology division of the Escola Paulista de Medicina/Federal University of São Paulo between 2000 and 2012 were retrospectively studied. Written Informed consent were obtained from all the participants.

The patients included in the study had a diagnosis of HCC based on radiological and/or histological criteria.

This study was retrospective and based on a review of the medical records of HCC patients. The prognostic systems chosen were then applied to the patients, and the results of each system were compared. Follow-up was censored on December 30, 2012.

### Clinical, demographic, and laboratory evaluations

The clinical analysis conducted provided detailed clinical histories and physical examinations. The following variables were included at the time of diagnosis: age, gender, aetiology of liver disease, CTP classification, Milan criteria, and presence or absence of liver cirrhosis.

Laboratory tests conducted included serum levels of albumin (g/dL), alpha-fetoprotein (AFP = IU/mL), aspartate aminotransferase (AST = U/L), alanine aminotransferase (ALT = U/L), alkaline phosphatase (AP = U/L), gamma glutamyl transferase (GGT = U/L), total bilirubin (TBR = mg/L), international normalized ratio (INR), platelets (n/μL), and creatinine (mg/dL).

Viral markers, such as HBsAg, anti-HBc, HBeAg, anti-HBe, and antiHCV, were tested using commercial enzyme-linked immunosorbent assays (ELISA). Hepatitis B virus (HBV) DNA levels were assessed by reverse transcription polymerase chain reaction (RT-PCR) with a detection limit of 20 IU/mL (Cobas TaqMan HBV, Roche Diagnostics), and HCVRNA levels were assessed by RT-PCR with a detection limit of 50 IU/mL.

The presence of liver cirrhosis was confirmed by histological analysis and/or clinical-laboratory parameters, such as ascites, hepatic encephalopathy, portal hypertension (splenomegaly, oesophageal varices, and thrombocytopenia), and ultrasonographic signs suggestive of liver cirrhosis. Cirrhosis caused by alcohol was considered for patients who reported a consumption of ethanol > 40 g/day for women and > 60 g/day for men over more than 5 years. Patients with liver cirrhosis were evaluated according to the CTP classification and the model for end-stage liver disease (MELD) score.

### Functional analysis

Patients were assessed for functional capacity on a scale based on the criteria of Karnorfsky (KPS) and the Eastern Cooperative Oncology Group (ECOG) [8]. For patients without KPS assessment documentation, scores were deduced as follows: ECOG 0 = KPS 100%, ECOG 1 = KPS 80–90%, ECOG 2 = KPS 60–70%, ECOG 3 = KPS 40–50%, and ECOG 4 = KPS 10–30%.

### Diagnosis of HCC

The diagnosis of HCC was based on computed tomography (CT) or magnetic resonance imaging (MRI) examinations of the liver that showed hypervascular lesions in the arterial phase and wash-out in the equilibrium phase. Indefinite lesions were subjected to image-guided biopsy. Tumours were evaluated according to size, number, tumour macrovascular invasion, and distant metastasis. The latter was screened by chest/abdominal tomography and bone scintigraphy.

### Treatment of HCC

Tumour treatment, when indicated, included liver transplantation (LT), surgical resection, tumour arterial chemoembolization (TACE), radiofrequency ablation (RF), and the administration of sorafenib. The Milan criteria were used to indicate the need for liver transplantation. The Milan and MELD criteria were applied to patients prior to the implementation of these criteria in Brazil in 2006.

### Analysis of prognostic systems

The prognostic systems used to evaluate survival were Okuda [9], CLIP [10], CUPI [11], GRETCH, [12] JIS score, modified JIS-TNM + alpha-fetoprotein system [14], TNM [15], and BCLC [16]. The interpretation of these systems is based on the sum of the numerical scores of their variables, in which higher numbers indicate greater severity.

### Statistical analysis

Results are expressed as the mean ± SD, median, and relative frequencies (%). For the analysis of survival, the variable *Status* was considered, which indicates whether death occurred, and the variable time of survival (in months) was used. Non-parametric techniques, such as the Kaplan-Meier Estimator and the log-rank comparison test, were also used. To estimate the multivariate model and to determine the hazard ratio, the Cox Backward Stepwise semi-parametric regression model was applied. Before applying the Cox regression model, the Spearman correlation test was used to evaluate the degrees of correlation between variables since significant correlations can generate problems of collinearity, therefore presenting errors in the interpretation of the coefficients of the regression model to be estimated. If the Spearman test showed a significant correlation between variables, Cox univariate regression models or size reduction techniques were applied. To identify which model exhibited the best performance at determining survival among the studied patients, the model fit measurements log-likelihood and Akaike Information Criterion (AIC) were used. Models with the highest log-likelihood and the lowest AIC values were considered the best models for predicting survival. The significance level used was *P*<0.05. SPSS 20 software (Chicago, IL, USA) was used for the statistical analyses.

## Results

### Patient characteristics

A total of 247 patients diagnosed with hepatocellular carcinoma followed in the liver transplantation section of the surgical gastroenterology division of the Escola Paulista de Medicina /Federal University of São Paulo between 2000 and 2012 were retrospectively studied. Among these patients, the mean age was 60 ± 10 years, with a predominance of men (74%). The main aetiology of chronic liver disease was HCV infection (73%), followed by alcoholic liver disease (12%), HBV infection (8%), and other aetiologies (7%). Liver cirrhosis was present in 92% of patients. These patients were classified into Child A (57%), B (36%), and C (7%) groups. These characteristics are represented in Table 2 and have been published previously. The mean number of nodules was 2 ± 1, ranging from 1–7 nodules, and the mean nodule size was 5 ± 3 cm in diameter, ranging from 0.9–21 cm. Distant metastases and macrovascular tumour invasion were found in 10% and 13% of cases, respectively. A total of 43% of the patients met the Milan criteria. The mean, median and range of serum AFP were 4.4 ± 13.7 IU/mL, 57 IU/mL and 1–102.436 IU/mL, respectively. (Table 3).

**Table 2:** Clinical and epidemiological characteristics of HCC patients (n = 247).

Clinical/epidemiological characteristics	
**Age (years)**	60±10 years
**Gender (%)**	
**Male**	182 (74)
**Female**	65 (26)
**Aetiology (%)**	
**Hepatitis C**	179 (73)
**Alcohol**	31 (12)
**Hepatitis B**	19 (8)
**Other**	18 (7)

**Table 3:** Characteristics of HCC tumours (n = 247).

Tumour characteristics	n = 247
**Number of nodules**	2±1
**Size of nodules**	5±3
**Milan criteria (%)**	
**Yes**	107 (43)
**No**	140 (57)
**Regional macrovascular invasion**	31 (13)
**Metastasis (%)**	
**Regional lymph node**	24 (10)
**Lung**	12 (50)
**Bone**	8 (33)
**Alpha-fetoprotein (IU/mL)**	4 (17)
**Mean**	4.4 ± 13.7 IU/mL
**Median**	57
**Range**	1–102.435
**IQR**	987

From the total sample of patients with HCC (n = 247), liver transplantation (LT) was performed in 10% of cases, surgical resection in 5%, transarterial chemoembolization (TACE) in 49%, radiofrequency (RF) ablation and percutaneous ethanol injection (PEI) in 1%, and Sorafenib was given to 14% of the patients (Table 4).

**Table 4:** Treatment of HCC patients (n = 247).

Treatment	n = 247 (%)
**Liver transplantation**	25 (10)
**Liver resection**	13 (5)
**TACE**	122 (49)
**RF**	3 (1)
**PEI injection**	5 (2)
**Sorafenib**	35 (14)
**Symptomatic treatment**	68 (28)

### Survival analysis

**Fig 1:**
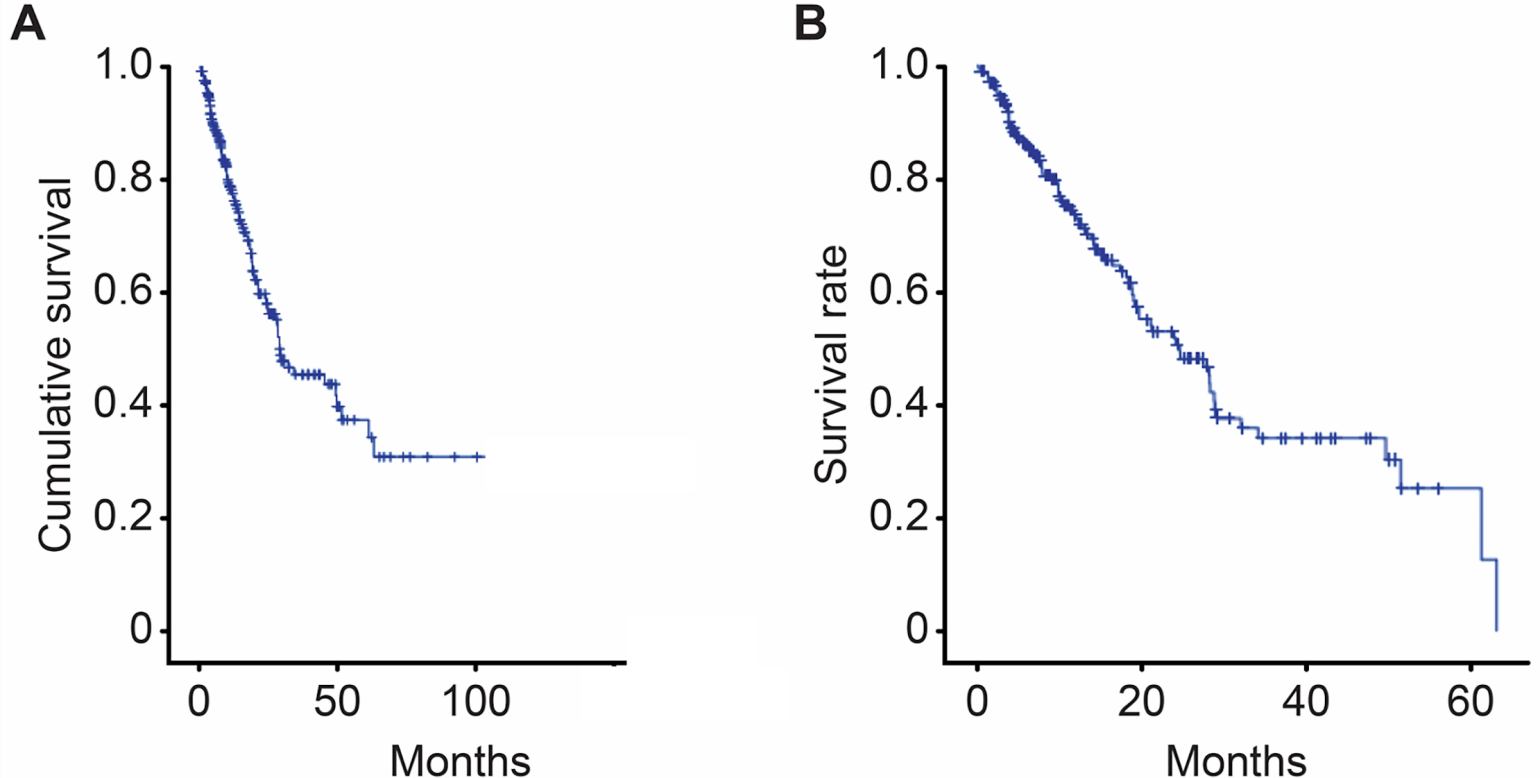
Survival Curve of the Studied Sample (n = 247) (A) and overall survival curve of the HCC patients (excluding those undergoing liver transplantations or surgical tumour resection) (n = 206) (B).

The overall survival time among the studied patients was, on average, 60 months, with 1-, 3-, and 5-year survival probabilities of 74%, 40%, and 26%, respectively (Fig 1). The median time from HCC diagnosis to the end of follow up or death, was 68 months.

**Fig 2:**
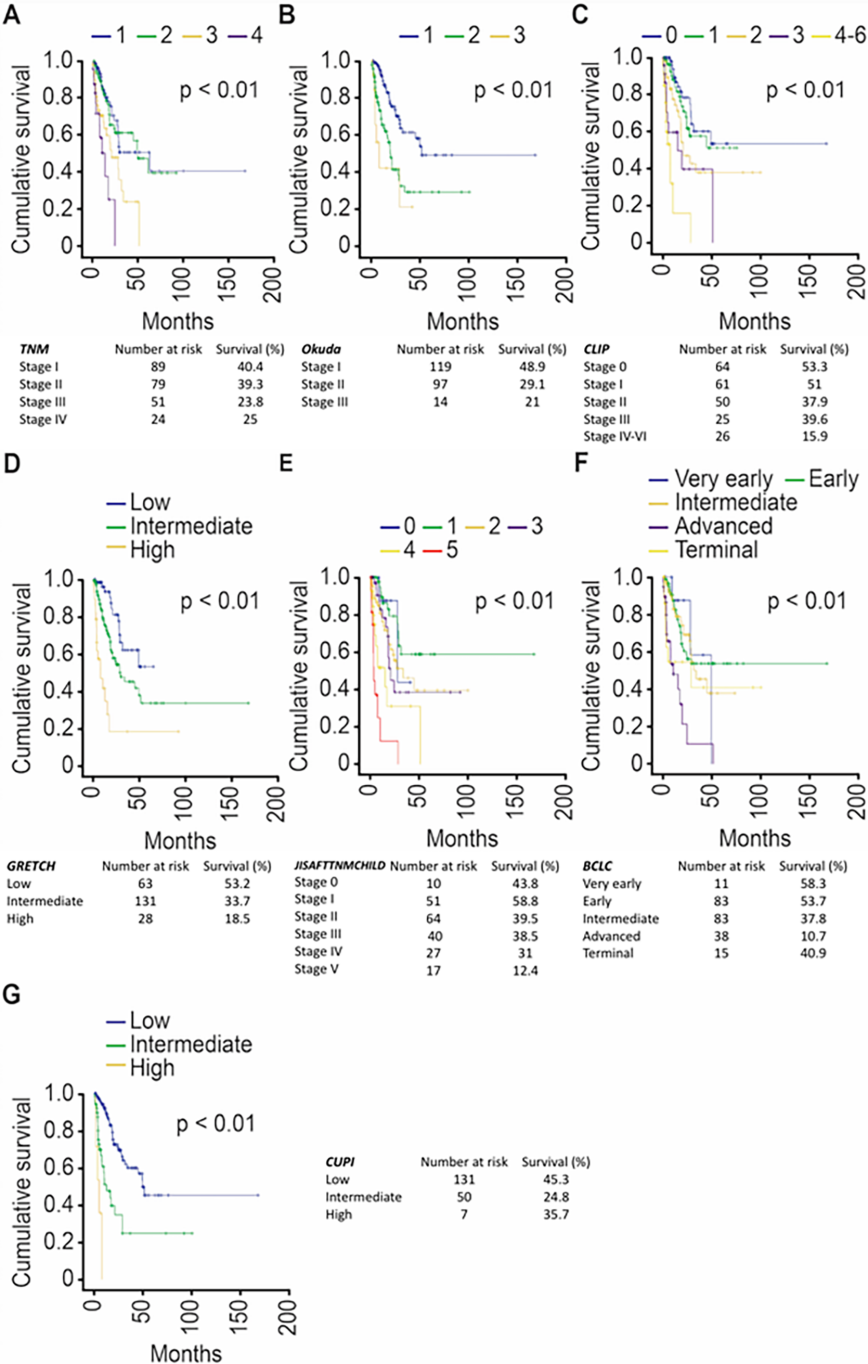
Survival curve for the variables TNM (A), Okuda (B), CLIP (C), GRETCH (D), JIS-AFP-TNM-CHILD (E), BCLC (F), and CUPI (G).

The curves of survival according to the systems studied are demonstrated in Fig 2. During our analysis of the best model for survival prediction, we found that the CUPI system exhibited the highest log-likelihood and the lowest AIC values (Table 5).

**Table 5:** Akaike information criterion and log-likelihood values for the analysed variables.

Variable	AIC	Log Likelihood
**Okuda**	729.285	−362.643
**BCLC**	742.897	−367.448
**JISAFTTNMCHILD**	665.767	−327.883
**CLIP**	706.536	−349.268
**CUPI**	566.480	−281.240
**GRETCH**	699.896	−347.948
**TNM**	802.962	−398.481

## Discussion

Unlike other solid malignant tumours, the outcome of hepatocellular carcinoma does not depend solely on the stage of the disease and the histological degree of differentiation but also on the liver disease underlying the neoplasm. Thus, the prognostic evaluation of HCC is complex and heterogeneous, which has led to the development of prognostic systems capable of guiding and improving the management of HCC patients [6,7]. Several prognostic classifications for HCC have been proposed that include the use of clinical, laboratory, radiological, and even molecular variables. However, no tumour staging system has proven to be ideal or been universally approved.

In a comparative analysis of various prognostic systems conducted by the present study, the TNM, Okuda, CLIP, GRETCH, modified JIS, and BCLC systems exhibited good correlations with each other and were individually important in the prediction of survival among the studied patients. However, in the final analysis, only the CUPI exhibited the best predictive performance.

The TNM staging system described by Pierre Denoix in 1943 and revised several times since is considered the most traditional staging system for solid tumours [15]. Despite its ease of use and usefulness in the surgical setting, TNM may not be sufficient to stage HCC, as it addresses only the peculiarities of the tumour and does not consider residual liver function, a factor that is often decisive for the prognosis of these patients [17–19].

The system proposed by Okuda et al. [20] in 1984 was the first to attempt to combine the degree of liver disease with tumour characteristics [9,20]. Patients were classified based on tumour volume and extent and on liver function data [20,21]. Although it is still used, the major criticism of the Okuda system is its lack of specific classifications and difficulty in categorizing tumour extent (less than or greater than 50% of the liver), in addition to the fact of not assessing vascular tumour extension and distant metastasis, two very important factors in the outcome of patients [22,23]. In the present study, it was possible to discriminate survival between the 1x2 and 1x3 scales, but not between 2x3, that is, it was not possible to discriminate the survival between those patients considered with moderately advanced tumours from those with very advanced tumours. This result points to a flaw in this system, which likely requires further stratification of its scales.

CLIP was first proposed in 1998 and was internationally validated in 2000 [10,21,24]. The superiority of CLIP over Okuda as a staging system was confirmed by Llovet et al. [25] in a clinical trial of 196 patients. However, as it was originally based upon a cohort of patients with advanced tumours, it is generally accepted that the CLIP system is more appropriate for patients with advanced disease and under palliative treatment. This statement was confirmed by Cillo et al. [26] with a cohort of 187 patients who underwent surgical treatment or ablation, in which CLIP did not exhibit a good correlation with mortality [26,27]. Another limitation of the CLIP lies in the evaluation of the tumour morphology, which, similar to the Okuda system, considers tumour extension as smaller or greater than 50% and as "massive"–very subjective terms. In the present study, it was not possible to find a significant difference between several scales of the CLIP, which greatly compromised the evaluation of this system in the prediction of survival of the patients studied.

Similar to CLIP, the French system proposed in 1999 by GRETCH classifies HCC patients based on radiological, laboratory, and clinical criteria [12]. Despite the ease of obtaining the necessary data for classification, the French staging system did not exhibit good prognostic capacity in the current study, as it does not use the size and number of tumours in its classification, which are important data for therapeutic decision-making in the study population. In addition, GRETCH considers portal thrombosis through ultrasound, which is of questionable accuracy compared with other imaging methods. Another limitation of this system is the fact that it has been validated in a large population that did not receive any type of treatment, which differs from the present study, where most received some type of treatment, including curative treatments, such as liver transplantation, resection, and radiofrequency ablation. In fact, although the GRETCH system in the present study presented a significant difference between the categories (low risk x intermediate risk x high risk of death) in relation to the determination of survival, this system did not present the greatest discrimination in relation to the other systems in the final analysis.

The JIS system was created in 2003 by the Liver Cancer Study Group of Japan (LCSGJ) and was later improved upon and modified in TNM (JIS-TNM-Child) and JIS-TNM-Child plus alpha fetoprotein (JISTNMAFPChild). In the present study, JISTNMAFPChild did not adequately discriminate survival between their scales and did not discriminate survival better when compared with the other systems. Thus, JIS was not well correlated with survival in either our analysis or several other studies, including a cohort study with 239 patients described by Marrero et al. [28] and a study by Zhang et al. [29] with 196 HCC patients.

The BCLC was developed and published in 1999 to stratify HCC into 4 stages and to provide a treatment algorithm for each stage based on variables related to tumour stage, liver function, and the functional status of the patient [16,30]. The BCLC was widely validated as the best and most comprehensive staging system for hepatocellular carcinoma in several studies [26–28,30–33]. In addition, it has the support of the European Association for the Study of the Liver (EASL) [34] and of the American Association for the Study of Liver Diseases (AASLD) [35] as the main tools for the prognostic definition of HCC. Despite all the advantages of BCLC, in the present study, the survival time could not be differentiated between some scales of the system (very early x intermediate or early x intermediate), which would compromise the therapeutic indications for a large number of the patients studied. In addition, the BCLC's discriminatory capacity to predict survival was lower than that of the other evaluated systems.

Several disadvantages of the BCLC have been reported, including restricted therapeutic options, especially in the intermediate stages, which represented an important number of patients in our study [36,37]. In addition, the BCLC system has been criticized by surgeons, who attest that the algorithm excludes patients who could benefit from curative resection, which is reserved only for the very early stages.

The CUPI was formulated by a Chinese group in 2002, through a refinement of the TNM system combined with prognostic factors applied in a multivariate Cox model [11]. Six clinical and laboratory variables are considered, and patients are divided into 3 stages of staging (low risk, intermediary risk, and high risk of death). The CUPI performed satisfactorily in several subsequent studies compared with other prognostic classification systems, as demonstrated by Shao et al. [38] in a study of 157 patients, by Li et al. [39] with 208 patients, and later in a study by Zhang et al. [29].

The CUPI is also an easy-to-use and well-designed scale that includes important data regarding the natural history of HCC as TNM, symptoms, ascites, alpha fetoprotein, bilirubin, and alkaline phosphatase [40,41]. Such variables appear to more broadly encompass HCC patients of any aetiology and of any stage.

In the present study, among the evaluated systems, The CUPI was the only system that showed significant difference in survival time according to the stratification of its categories (low risk x intermediate risk, low risk x high risk, intermediate risk x high risk). In addition, it presented the best performance in predicting survival with the lowest AKAIKE and the highest log likelihood value.

Although CUPI was validated in a cohort predominantly consisting of hepatitis B virus carriers, its performance in our study with a predominance of HCV was very good. In addition, although CUPI had a cohort with a majority of patients with advanced tumours, this fact also did not compromise its performance in our sample, which was composed of a very heterogeneous population and with few cases with advanced tumours.

Regarding the aetiological difference in liver disease between the population from the Chinese study and our study, the superiority of the CUPI was recently reaffirmed in a large international cohort study conducted by Chan et al.[42] with the participation of one Chinese group (517 patients, 80% with HBV) and one British group (567 patients, 92% with HCV), in which the authors demonstrated the applicability of the CUPI in Western populations, in which hepatitis B is not predominant [42].

It is worth highlighting some limitations of the present study, such as the retrospective nature of the study, its development in a single care centre and demographic differences in the clinical and epidemiological parameters of HCC, which may hinder the generalization and applicability of results in other Brazilian regions. Despite these limitations, this study included a significant number of patients with sufficient clinical data and a broad spectrum of early, intermediate, and advanced lesions.

Although the CUPI system has been demonstrated to be the most appropriate HCC staging system for the studied population, the choice of an ideal system is a controversial subject, and future studies with larger numbers of patients will be necessary to validate the CUPI as a method of choice in other populations.

## Supporting information

S1 Database (XLS)
